# Exploring the Influence of Sex and Gender on Cancer: A Comprehensive Analysis

**DOI:** 10.1002/cam4.71263

**Published:** 2025-09-27

**Authors:** Teresa Gisinger, Alexandra Kautzky‐Willer, Alexander Kautzky

**Affiliations:** ^1^ Unit of Gender Medicine, Division of Endocrinology and Metabolism, Department of Internal Medicine III Medical University of Vienna Vienna Austria; ^2^ Department of Psychiatry and Psychotherapy, Clinical Division of Social Psychiatry Medical University of Vienna Vienna Austria; ^3^ Department of Clinical Neuroscience, Division of Insurance Medicine Karolinska Institute Solna Sweden

**Keywords:** biological differences, cancer risk, gender medicine, gender‐related variables, sex differences

## Abstract

**Purpose:**

Our aim was to untangle the effect of biological sex and psychosocial gender on rates of self‐reported cancer.

**Methods:**

For this study, the Austrian Health Information Survey 2019 was used. Individuals with cancer (*n* = 415) were selected and divided by sex (female 53.3%). Descriptive results were reported as mean/standard deviation or frequency/percentage. Next, the cancer versus non‐cancer and female versus male cancer cohorts were compared by using a chi‐square test or an unpaired *t*‐test. By logistic regression models adjusted by age, the association of cancer, biological sex, and gender‐related variables (including employment status, educational level, marital status, household income and size) was investigated in the total cohort, as well as stratified by sex.

**Results:**

Self‐reported cancer was positively related with being unemployed/retired versus employed (OR 2.06, *p* < 0.001), being married versus single (OR 1.90, *p* = 0.005), household size over 1 compared to 1 (OR 1.42, *p* = 0.025), self‐reported depression (OR 2.13, *p* < 0.001), and former smoking (OR 1.84, *p* < 0.001). A negative relation between having cancer and being a daily smoker was seen (OR 0.22, *p* = 0.006). No association between cancer and biological sex was found. Similar correlations of gender‐related variables on cancer rates were found in the sex‐disaggregated analyses; however, marital status as well as daily smoking were not associated with cancer diagnosis in males. In contrast to males, in females former smoking and alcohol consumption showed no association with cancer rates.

**Conclusion:**

Our findings suggest that gender‐related variables, rather than biological sex, are associated with sel‐reported cancer.

## Graphical abstract

Psychosocial gender is related to rates of self‐reported cancer. Especially, conditions involving significant caregiving responsibilities and stress are correlated with higher rates of self‐reported cancer.

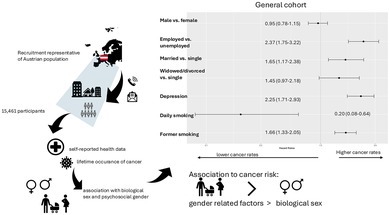

## 1 Background

In 2021, cancer was reported as the second leading cause of death across European Union member states [1]. Sex differences were observed in cancer prognosis and outcome, and with few exceptions, higher cancer prevalences were reported in men than women [2, 3]. Sex‐specific differences in risk factors such as dietary behavior, smoking, and alcohol consumption contribute to these gaps [4]; however, the risk for cancer remained higher in males compared to females also when adjusting for these factors [5]. Along these lines, males have a higher incidence and lower survival in pediatric cancers even though risk behaviors such as smoking are not yet present [6]. As a result, biological factors—like sex‐specific gene expression—are thought to contribute to differences in oncogene activity, which can affect cancer risk [7].

Next to biological sex, psychosocial gender affects the risk and outcome of different diseases [8]. Gender is closely related to socioeconomic variables such as educational level, employment status, marital status, and household income [9]. Lower educational level as well as income was associated with a higher risk of cancer [10], putatively mediated by a lower frequency of preventive care examination [11]. However, few studies investigated the effect of psychosocial gender on cancer risk [12]. Currently, no study assessed the relation of cancer risk to both biological sex and different gender‐related variables.

The aim of this study is to associate gender‐related variables such as educational level, marital status, employment status, household size, and income with rates of self‐reported cancer. Therefore, gender‐related variables were compared between males and females with cancer. Finally, associations of gender‐related variables with rates of self‐reported cancer were assessed separately among females and males. This integrated view on biological sex and psychosocial gender aims at better characterizing individuals at risk for cancer to allow personalized cancer screening and to improve efficiency and outcomes in healthcare systems.

## 2 Materials and Methods

### 2.1 Study Design

Data from the Austrian Health Information Survey 2019 (AT‐HIS) was used. Participants had to be over 15 years old and had to live in Austria. A total of 15,461 study participants were chosen to represent the Austrian population. Beforehand, written consent from each participant was gained by Statistik Austria. The data was obtained from Statistik Austria after an application with a project proposal. Ethics approval was gained from the ethic board of the Medical University of Vienna (1859/2019).

### 2.2 Study Cohorts and Disease Variables

Within this database, cancer and comorbidities were self‐reported. Whether individuals suffered from cancer was asked as a binary‐response question “Do you have any malignant disease in the last 12 months?” Based on this question, individuals were further stratified by presence (*n* = 415) or absence (*n* = 15,046) of cancer diagnoses. The group with cancer was further split by biological sex (male: *n* = 194, female: *n* = 221).

### 2.3 Outcome Measures

The dataset was reviewed for gender‐related and well‐established cancer risk factors. Educational level, employment status, marital status, household size, and household income were defined as gender‐related variables. Educational level was dichotomized as low/middle and high level. Employment status was originally reported as employed, unemployed, retired, and in training. Due to low frequencies of the minority outcomes, employment status was dichotomized to active employment vs. no active employment (in training, unemployment, or retirement). Marital status was divided into being single, being married/common‐in‐law, being widowed, and being divorced. Lastly, household income was reported in quintiles. Cancer‐related risk variables were defined as the binary former smoking and daily smoking variable. Moreover, consuming alcohol more than twice and four times a week was considered as a cancer‐related risk variable. Presence or absence of self‐reported depression within the last 12 months was also included. Lastly, preventive care usage was evaluated with the questions of whether individuals aged 50 years or older underwent a fecal occult blood test or colonoscopy in the last 5 years. Females aged 50 years or older, were asked whether they had a mammography in the last 12 months. From all females regardless of age, the usage of a PAP smear in the last 12 months was inquired.

### 2.4 Statistical Analysis

Descriptive results were reported as means and standard deviations for continuous variables. Categorical variables were reported as frequencies and percentages. Further, group differences according to sex and self‐reported cancer were compared by chi‐square tests for ordinal and unpaired t‐tests for numerical variables. Logistic regression models were computed with self‐reported cancer as the outcome variable and cancer‐ and gender‐related variables as predictors, and are age adjusted. The analyses were weighted to the general population. As fecal occult blood testing, colonoscopy, and mammography are recommended in older age, the frequency of these preventive care examinations was added to an additional logistic regression model done in individuals aged 50 years and older.

## 3 Results

### 3.1 Comparing Individuals With and Without Cancer

In Table 1 cohort baseline characteristics are reported, stratified by presence or absence of cancer as well as sex. Unemployment/retirement was more frequent in individuals with cancer compared to those without cancer (81.7% vs. 47.9%, *p* < 0.001). Moreover, individuals with cancer had higher rates of low or moderate educational level (77.8% vs. 72.4%, *p* = 0.017), were more often married (married/common‐in‐law: 84.1% vs. 54.5%, single: 10.4% vs. 31.6%, widowed/divorced: 25.8% vs. 16.7%, *p* < 0.001), had lower household size (mean 2.09 vs. 2.62, *p* < 0.001), higher rates of low income (low: 25.8% vs. 22.1%, middle: 65.3% vs. 65.3%, high: 8.9% vs. 15.3%, *p* < 0.001), lower rates of age < 55 years (18.3% vs. 57.1%, < 0.001), higher body mass index (BMI) (< 24.9 kg/m^2^: 40.2% vs. 48.7%, 25.0–29.9 kg/m^2^: 39.5% vs. 35%, > 30.0 kg/m^2^: 20.2% vs. 16.3%, *p* = 0.002), higher rates of self‐reported depression (15.7% vs. 6.8%, *p* < 0.001), lower rates of daily smoking (11.1% vs. 18.6%, *p* < 0.001), and higher rates of former smoking (40.2% vs. 27.8%, *p* < 0.001) compared to individuals without cancer. Higher usage of preventive care measures such as fecal occult blood test and colonoscopy was reported in the population over the age of 50 years with cancer compared to those without cancer (fecal occult blood test: 65.6% vs. 54.7%, *p* < 0.001; colonoscopy: 54.4% vs. 40.5%, *p* < 0.001). In the female cohort, higher rates of mammography in the cancer cohort were found compared to the no‐cancer cohort (58.9% vs. 34.6%, *p* < 0.001).

**TABLE 1:** Basic characteristics.

Variable	No cancer (*n* = 15,046)	Cancer (*n* = 415)	Chi square/unpaired *t*‐test (no cancer vs. cancer)	Cancer, male (*n* = 194)	Cancer, female (*n* = 221)	Chi square/unpaired *t*‐test (male vs. female)
Sex			0.909	…	…	…
Male	6972 (46.3%)	194 (46.7%)				
Female	8074 (53.7%)	221 (53.3%)				
Education level			0.017			0.724
Low/middle	10,892 (72.4%)	323 (77.8%)		149 (76.8%)	174 (78.7%)	
High	4154 (27.6%)	92 (22.2%)		45 (23.2%)	47 (21.3%)	
Employment status			< 0.001			0.011
Employed	7837 (52.1%)	76 (18.3%)		25 (12.9%)	51 (23.1%)	
Unemployed	7209 (47.9%)	339 (81.7%)		169 (87.1%)	170 (76.9%)	
Employment status			< 0.001			< 0.001
Employed	7913 (52.6%)	76 (18.3%)		25 (12.9%)	51 (23.1%)	
Unemployed	1589 (10.6%)	39 (9.4%)		9 (4.6%)	30 (13.6%)	
Retired	4985 (33.1%)	295 (71.1%)		158 (81.4%)	137 (62.0%)	
In training	974 (6.5%)	5 (1.2%)		2 (1.0%)	3 (1.4%)	
Work hours						
Full‐time	2079 (13.8%)	26 (6.3%)	0.166	1 (0.5%)	25 (11.3%)	< 0.001
Part‐time	5758 (38.3%)	50 (12.1%)		24 (12.4%)	26 (11.8%)	
Marital status			< 0.001			< 0.001
Single	4756 (31.6%)	43 (10.4%)		17 (8.8%)	26 (11.8%)	
Married/common‐in‐law	8197 (54.5%)	265 (84.1%)		153 (78.9%)	112 (50.7%)	
Widowed/divorced	2508 (16.7%)	107 (25.8%)		24 (12.4%)	83 (37.6%)	
Household size (mean ± standard deviation)	2.62 (1.33)	2.09 (0.95)	< 0.001	2.14 (0.89)	2.04 (1.01)	0.290
Household size 1	2917 (19.4%)	92 (22.2%)	0.177	29 (14.9%)	63 (28.5%)	0.001
More than 1	12,129 (80.6%)	323 (77.8%)		165 (85.1%)	158 (71.5%)	
Household income			< 0.001			0.178
Low	3329 (22.1%)	107 (25.8%)		42 (21.6%)	65 (29.4%)	
Middle	9830 (65.3%)	271 (65.3%)		135 (69.6%)	136 (61.5%)	
High	2302 (15.3%)	37 (8.9%)		17 (8.8%)	20 (9.0%)	
Age group			< 0.001			< 0.001
< 55	8587 (57.1%)	76 (18.3%)		22 (11.3%)	54 (24.4%)	
55–64	2695 (17.9%)	108 (26.0%)		44 (22.7%)	64 (29.0%)	
65–74	1952 (13.0%)	86 (20.7%)		50 (25.8%)	36 (16.3%)	
74–84	1376 (9.1%)	107 (25.8%)		61 (31.4%)	46 (20.8%)	
> 85	436 (2.3%)	38 (9.2%)		17 (8.8%)	21 (9.5%)	
BMI			0.002			< 0.001
< 24.9 kg/m^2^	7333 (48.7%)	167 (40.2%)		61 (31.4%)	106 (48.0%)	
25.0–29.9 kg/m^2^	5263 (35.0%)	164 (39.5%)		98 (50.5%)	66 (29.9%)	
> 30.0 kg/m^2^	2450 (16.3%)	84 (20.2%)		35 (18.0%)	49 (22.2%)	
Depression						
General	1027 (6.8%)	71 (15.7%)	< 0.001	33 (17.0%)	38 (17.2%)	0.990
Male	337 (4.8%)	…				
Female	690 (8.5%)	…				
Daily smoking	2801 (18.6%)	46 (11.1%)	< 0.001	16 (8.2%)	30 (13.6%)	0.211
Former smoker	4184 (27.8%)	167 (40.2%)	< 0.001	110 (56.7%)	57 (25.8%)	< 0.001
Alcohol consumption more than twice a week	2714 (18.0%)	78 (18.8%)	0.741	56 (28.9%)	22 (10.0%)	< 0.001
Alcohol consumption more than four times a week	1315 (8.7%)	43 (10.4%)	0.288	31 (16.0%)	12 (5.4%)	< 0.001
Fecal occult blood testing in the last 5 years in population over 50 years	4322 (54.7%)	240 (65.6%)	< 0.001	126 (69.6%)	114 (61.6%)	0.134
Colonoscopy in the last 5 years in population over 50 years	3203 (40.5%)	199 (54.4%)	< 0.001	110 (60.8%)	89 (48.1%)	0.020
Mammography in the last year in population over 50 years	1477 (34.6%)	109 (58.9%)	< 0.001	…	109 (58.9%)	…
PAP test in the last year	1762 (50.2%)	125 (56.6%)	0.073	…	125 (56.6%)	…

*Note:* The frequency or mean and standard deviation for each variable in the general and cancer cohort. Furthermore, the same values are reported for the male and female cohort. Lastly, the characteristics of the non‐cancer versus cancer and male versus female cohorts are compared by a chi‐square test for ordinal and an unpaired *t*‐test for numerical variables and the *p* values are reported here.

### 3.2 Comparing Male and Female Individuals With Cancer

Females with cancer were more likely employed (23.1% vs. 12.9%, *p* = 0.011), single (11.8% vs. 8.8%, *p* < 0.001), widowed or divorced (37.6% vs. 12.4%, *p* < 0.001), of an age under 55 years (24.4% vs. 11.3%, *p* < 0.001), normal weight BMI (48.0% vs. 31.4%, *p* < 0.001), and consumed alcohol less often alcohol (more than twice a week: 10.0% vs. 28.9%, *p* < 0.001; more than four times a week: 5.4% vs. 16.0%, *p* < 0.001; see Table 1) compared to males. Higher usage of colonoscopy in males with cancer was found compared to females (60.8% vs. 48.1%, *p* = 0.020).

### 3.3 Association of Sex and Gender‐Related Variables With Cancer

Biological sex was not associated with cancer (OR 0.93, *p* = 0.761, Table 2). Having cancer was positively related to being unemployed/retired (OR 2.06, *p* < 0.001), being married rather than single (OR 1.90, *p* = 0.005), household size over 1 (OR 1.42, *p* = 0.025), self‐reported depression (OR 2.13, *p* < 0.001), and former smoking (OR 1.84, *p* < 0.001). A negative relation between cancer and being a daily smoker was seen (OR 0.22, *p* = 0.006). A positive association of the usage of fecal occult blood tests or colonoscopy in the last 5 years and self‐reported cancer rates was seen (fecal occult blood test: OR 1.72, *p* < 0.001, colonoscopy: OR 1.95, *p* < 0.001).

**TABLE 2:** Associations of sex and gender with cancer in the total, male, and female cohort.

Variable	Total cohort		Male cohort		Female cohort	
	Odds ratio (95% confidence interval)	*p* value	Odds ratio (95% confidence interval)	*p* value	Odds ratio (95% confidence interval)	*p* value
Education level (middle/high vs. low)	0.94 (0.71–1.25)	0.683	0.76 (0.54–1.07)	0.121	1.29 (0.91–1.81)	0.141
Employment status (unemployed/retired vs. employed)	2.06 (1.42–2.98)	< 0.001	3.86 (2.34–6.52)	< 0.001	1.62 (1.11–2.38)	0.013
Work hours	0.94 (0.53–1.14)	0.835	2.66 (0.512–0.49)	0.355	0.98 (0.56–1.72)	0.952
Marital status						
Married vs. single	1.90 (1.22–2.97)	0.005	1.66 (0.97–3.00)	0.076	1.45 (0.93–2.35)	0.114
Widowed/divorced vs. single	1.50 (0.88–2.56)	0.141	1.11 (0.56–2.25)	0.759	1.75 (1.06–2.96)	0.032
Household size more than 1 vs. 1	1.42 (1.05–1.93)	0.025	1.38 (0.93–2.12)	0.119	1.09 (0.80–1.52)	0.584
Household income						
Middle vs. low	1.31 (0.97–1.76)	0.077 0.777	1.15 (0.81–1.67)	0.447 0.838	1.20 (0.88–1.65)	0.263 0.738
High vs. low	1.07 (0.67–1.72)		0.94 (0.51–1.67)		1.10 (0.63–1.84)	
Depression	2.13 (1.51–2.99)	< 0.001	3.52 (2.32–5.23)	< 0.001	1.79 (1.23–2.55)	0.002
BMI						
25–29.9 vs. < 25	1.00 (0.76–1.31)	0.996	1.13 (0.81–1.58)	0.466	0.98 (0.71–1.34)	0.907
> 29.9 vs. < 25	1.20 (0.87–1.66)	0.264	1.03 (0.66–1.58)	0.889	1.39 (0.97–1.97)	0.064
Daily smoking	0.22 (0.08–0.65)	0.006	0.34 (0.08–2.45)	0.200	0.11 (0.03–0.62)	0.003
Former smoker	1.84 (1.42–2.38)	< 0.001	1.93 (1.41–2.66)	< 0.001	1.27 (0.91–1.75)	0.154
Alcohol consumption more than twice a week	1.05 (0.78–1.41)	0.750	1.38 (1.01–1.92)	0.048	1.05 (0.68–1.69)	0.830
Alcohol consumption more than four times a week	1.05 (0.72–1.52)	0.810	1.55 (1.06–2.35)	0.031	0.96 (0.55–1.85)	0.902
Biological sex	0.93 (0.76–1.23)	0.761	…	…	…	…
Fecal occult blood testing in the last 5 years	1.72 (1.32–2.23)	< 0.001	1.81 (1.32–2.53)	< 0.001	1.43 (1.06–1.94)	0.022
Colonoscopy in the last 5 years	1.95 (1.49–2.55)	< 0.001	2.01 (1.48–2.74)	< 0.001	1.47 (1.09–1.98)	0.010
Mammography in the last year	…	…	…	…	3.33 (2.44–4.57)	< 0.001
PAP test in the last year	…	…	…	…	2.12 (1.58–2.86)	< 0.001

*Note:* The relation of gender‐related variables with self‐reported cancer in the general, male and female cohort is described by logistic regression. Here the Odds Ration, 95% confidence interval and *p* values are reported.

Analyses were repeated after stratification by sex (see Table 2). Among both sexes, but more so in men than women, being unemployed/retired (females: OR 1.62, *p* = 0.013; males: OR 3.86, *p* < 0.001) and depression (females: OR 1.79, *p* = 0.002; males: OR 3.52, *p* < 0.001) were associated with cancer. The usage of preventive care examinations was associated with a higher rate of self‐reported cancer in both males and females. Nevertheless, a stronger association was found in males (fecal occult blood test males: OR 1.81, *p* < 0.001, females: OR 1.43, *p* = 0.022, colonoscopy males: OR 2.01, *p* < 0.001, females: OR 1.47, *p* = 0.010). Former smoking (OR = 1.93, *p* < 0.001) and alcohol consumption, both more than twice and four times a week (OR 1.38, *p* = 0.048 and OR 1.55, *p* = 0.031), were associated with cancer only in men. Only among women, self‐reported cancer was positively associated with being widowed or divorced compared to single (OR 1.75, *p* = 0.032) and negatively with daily smoking (OR = 0.11, *p* = 0.003). In females, the usage of mammography or PAP smear within the last 12 months was associated with a higher cancer rate (mammography: OR 3.33, *p* < 0.001; PAP smear: OR 2.12, *p* < 0.001).

## 4 Discussion

In this Austrian cohort, gender‐related variables rather than biological sex showed associations with self‐reported cancer. Both shared and sex‐specific associations were found. Differences in gender‐related variables seen in individuals with cancer compared to those without cancer included higher rates of unemployment/retirement and lower rates of living single, also when adjusting for the higher age in the cancer cohort. Comparing both sexes, being widowed or divorced was only associated with cancer among women.

Education level is known to promote better preventive care access and can therefore reduce risk for cancer [11]. Contrary to previous reports of higher risk of cancer in people with lower educational levels [10, 13], here no association was found. This may be due to health care utilization being covered by public insurance in Austria [14]. Still, our findings show that preventive care examinations such as fecal occult blood tests and colonoscopy are associated with a higher rate of cancer in both sexes. However, the association was stronger in males, although females are more likely to attend preventive care examinations [15]. Furthermore, females with cancer and an age of 50 or older had a higher frequency of mammography compared to females with an age of 50 or older without cancer, but no difference in PAP smear frequency in females with and without cancer was found. It is suggested that lower educational levels are associated with a higher percentage of other cancer‐related risk factors, such as alcohol consumption or smoking [16]. However, the lack of an observed association between education level and cancer risk may be due to limitations in our study design. Firstly, educational level is only measured in two levels. Secondly, there may be a selection bias, as people with low educational levels are less likely to participate in the AT‐HIS health survey.

In this cohort, being unemployed/retired was correlated with higher rates of self‐reported cancer. Previous studies investigated that lower income is associated with higher risk for cancer, which is also related to being unemployed [10]. Nevertheless, in our study, household income had no association with increased rates of self‐reported cancer. As previously mentioned, most preventive care examinations in Austria are covered by public insurance and are therefore free of charge. This suggests that low household income may have a smaller impact on health outcomes in Austria compared to many other countries. Moreover, as seen in the basic characteristics of our study population, the majority of the participants are of older age and retired, and therefore, the household income might not influence the cancer rate. Previous work already reported that gender‐related variables, including household income, do not correlate with unmet healthcare in the used database, AT‐HIS [17]. In contrast, people with lower income and education might not be aware of the importance of preventive care examinations and therefore might be diagnosed with cancer less frequently compared to individuals with a higher educational level and income [18, 19].

Moreover, looking at the household composition, a lower risk for cancer in larger families was reported [20]. For a long time, family has been cited as a health‐promoting factor, and family size is suggested to be associated with life satisfaction [20]. It is postulated that in a bigger family, one family member might be responsible for reminding the others to have necessary medical examinations and a healthy lifestyle [21]. On the other hand, past literature analyzed a higher risk of cardiometabolic diseases in individuals with higher caregiving responsibilities [22]. For example, a married individual might have more responsibilities for providing care to other family members, including a spouse, children, and a parent‐in‐law compared to unmarried individuals [22]. Next, a higher stress level in people being employed besides their high caregiving responsibilities is also known [22]. In our study, no significant difference in household size between individuals with and without cancer was found. Nevertheless, a mild association of a household size over 1 compared to 1 and rates of self‐reported cancer was found, which is underlined by our finding that being married is associated with cancer compared to being single. The finding can, on one hand, be explained by the younger age of individuals without cancer, making them more likely to live in single‐person households, but potentially also by a higher stress level experienced when having to take care of multiple family members. Nevertheless, our analyses adjusted for age. Agreeing with previous literature, being widowed or divorced was associated with higher rates of self‐reported cancer. This could be caused by social isolation [13]. Moreover, it is known that people who are married are diagnosed with cancer at an earlier stage compared to people who are not married [23]. This finding could be caused by an engaged spouse who advises their partner to seek medical care or even to use the preventive care examinations [24]. Being widowed or divorced is a known risk factor for mental illness such as depression [25], a common mental illness that was also previously associated with increased risk for cancer [26]. A bidirectional relation between depression and cancer was proposed, considering that, on one hand, pre‐existing depression was associated with some [27] but not all types of cancer [28], and on the other hand, up to half of patients diagnosed with cancer also receive a diagnosis of depression within a few years [29]. While we cannot make assumptions on the directionality of the association, also here doubled rates of self‐reported depression were observed among patients with cancer. Interestingly, doubled OR were computed for men compared to women (3.52 vs. 1.79), suggesting that sex impacts the relationship between cancer and depression. Controversial findings are reported concerning sex differences in depression risk among cancer patients. A study in colorectal cancer patients reports a higher depression and anxiety rate in female patients compared to males, while a study of Austrian individuals with various cancer types could not find sex differences within the depression rate [30, 31]. The well‐established sex gap in depression, with higher prevalence in women (8.5% vs. 4.8%), was observed among individuals without cancer but was nearly closed among those with cancer (17.2% vs. 17.0%) [32]. Increased rates of depression as well as a lack of sex differences in depression prevalence agree with previous findings in palliative care but also community samples of individuals with cancer [33, 34]. A diagnosis of cancer may thereby outdo the effect of gender‐related factors contributing to increased rates of depression in women [32], leading to similarly high rates in women and men with cancer in this cohort.

Regarding cancer‐related factors among the total cohort, females less frequently consumed alcohol and were less likely former smokers compared to males. Further, females were more likely to have a lower BMI compared to males. Former smoking was related to having cancer in our study in the general and male cohort, but not in the female cohort, which could be explained by cancer risk and mortality in females being more strongly associated with passive than with active smoking [35]. In our study, daily smoking was negatively associated with cancer, indicating that individuals with cancer smoke less after diagnosis, which is in line with current literature [36]. Concerning alcohol consumption, previous literature reports that higher alcohol intake is related to higher cancer risk [37]. Nevertheless, an association between alcohol intake and having cancer was found only in the male study cohort. However, changes in drinking behavior reactive to a diagnosis of cancer may have contributed to inconsistent associations. Although our study does not specify the exact types of cancer, it is reasonable to assume that the majority of cases involve the most common forms—such as breast cancer in females, prostate cancer in males, as well as lung and colorectal cancers—which are, to varying degrees, associated with smoking and alcohol consumption.

Nevertheless, we see a higher female participation in the used survey. Females are often more likely to respond to health‐related surveys than males, a phenomenon well documented in survey research [38]. This sex imbalance can introduce bias, particularly in self‐reported outcomes and health service use, as women may report different symptoms, care patterns, or perceptions of care compared to men. Therefore, the used data was weighted to the population regarding the best practices in health survey methodology [39].

It is already known that obesity is related to various cancer types [40] and this association was suggested to be stronger in women [41]. In our study, higher BMI showed no association with cancer. However, study participants may have lost weight because of disease progression or therapy, masking associations between premorbid BMI and rates of self‐reported cancer.

Among individuals with cancer, females were more likely to be employed and single, divorced, or widowed. Currently, only a little scientific work compares gender‐related variables sex‐specifically in individuals with cancer. Nevertheless, past literature reports that the association of household size with cancer risk is stronger in males compared to females [22]. Stratifying the cohort by sex, sex‐specific associations with cancer were found. In particular, in females, being widowed or divorced was linked to increased rates of self‐reported cancer, whereas in males, no association was found. Moreover, males reported an association with former smoking, alcohol consumption, and cancer rates in contrast to females.

## 5 Limitations

Concerning limitations, our data is self‐reported, which could result in over‐ or underreporting. Second, our database did not specify the types of cancer, staging, or cancer duration. Furthermore, we incorporate socioeconomic variables as proxies for gender‐related factors, as previously acknowledged in retrospective studies [9]. However, the database lacks more specific gender‐related variables such as social norms, work‐related stress, family dynamics, and others [42]. Lastly, we experience a selection bias, which we cannot avoid in a retrospective study design.

## 6 Conclusion/Implications

Despite these limitations, to our knowledge, the novel aspect of this study is considering both biological sex and gender‐related variables when analyzing rates of self‐reported cancer. Our findings suggest that conditions involving significant caregiving responsibilities and stress, including unemployment/retirement, are correlated with cancer. Consequently, our study highlights the need for improved support systems for this vulnerable population in current clinical practice.

## Author Contributions

**Teresa Gisinger:** conceptualization (lead), formal analysis (lead), writing – original draft (lead). **Alexandra Kautzky‐Willer:** conceptualization (supporting), methodology (supporting), supervision (supporting), writing – review and editing (supporting). **Alexander Kautzky:** conceptualization (supporting), methodology (supporting), supervision (lead), writing – review and editing (lead).

## Ethics Statement

Ethics approval was gained from the ethic board of the Medical University of Vienna (1859/2019).

## Conflicts of Interest

The authors declare no conflicts of interest.

## Data Availability

The research data is confidential and can be accessed after application at Statistik Austria.
